# Primary intestinal diffuse large B-cell lymphoma: novel insights and clinical perception

**DOI:** 10.3389/fonc.2024.1404298

**Published:** 2024-08-15

**Authors:** Xiaojun Chen, Jing Wang, Yanquan Liu, Suxia Lin, Jianzhen Shen, Yue Yin, Yili Wang

**Affiliations:** ^1^ Department of Hematology and Rheumatology, The Affiliated Hospital of Putian University, Putian, Fujian, China; ^2^ Department of Oncology, The First Affiliated Hospital of Gannan Medical University, Jiangxi Clinical Research Center for Cancer, Ganzhou, Jiangxi, China; ^3^ Department of Hematology, The First School of Clinical Medicine, Guangdong Medical University, Dongguan, Guangdong, China; ^4^ Department of Hematology, Fujian Medical University Union Hospital, Fuzhou, Fujian, China

**Keywords:** diffuse large B-cell lymphoma, intestinal lymphoma, extranodal lymphoma, gastrointestinal tract, prognosis

## Abstract

**Background:**

Extranodal Non-Hodgkin lymphoma (NHL) is more prevalent in the gastrointestinal (GI) tract than in other sites. This study aimed to explore the clinical features and prognostic factors of primary intestinal diffuse large B-cell lymphoma (PI-DLBCL), in order to provide new references for basic research and clinical diagnosis and treatment of the rare extranodal malignant lymphoma.

**Methods:**

The clinical data of 88 patients with PI-DLBCL admitted to Fujian Medical University Union Hospital from June 2011 to June 2022 were retrospectively studied, the clinical and pathological features, diagnosis and treatment process and prognosis of PI-DLBCL were analyzed, and univariate and multivariate analysis of prognostic factors was carried out. The Kaplan-Meier method was used for survival analysis. Meanwhile, the latest literature from PubMed was retrieved to systematically discuss the research progress in the diagnosis and treatment of PI-DLBCL.

**Results:**

Among the 88 patients with PI-DLBCL included in this study, 60 cases were males (68.18%), 28 cases were females (31.82%), and 62 patients (70.45%) were complaining of abdominal pain, and the second most common clinical manifestation was changes in bowel habits in 16 (18.18%), with a median age of onset of 57 (17–82) years. The first-line treatment regimen was surgery combined with R-CHOP chemotherapy (56.82%). The median follow-up time was 72 (1–148) months, 51 (57.95%) of 88 patients with PI-DLBCL survived, 30 patients (34.09%) died, 7 patients (7.95%) were lost to follow-up, and the PFS rates of 1-year, 3-year and 5-year were 57.95%, 29.55% and 15.91%, and the OS rates of 1-year, 3-year and 5-year were 79.55%, 45.45% and 28.41%, respectively. The results of univariate *Cox* regression analysis showed that ECOG score, Lugano stage, B symptoms, IPI score, white blood cells, serum LDH, albumin, β_2_ microglobulin were the influencing factors of OS in PI-DLBCL patients, and ECOG score, Lugano stage, B symptoms, IPI score, white blood cells, serum LDH, albumin, β_2_ microglobulin were all the influencing factors of PFS in PI-DLBCL patients. The results of multivariate *Cox* analysis showed that Lugano stage may be an independent prognostic factor affecting OS and PFS in PI-DLBCL patients.

**Conclusion:**

PI-DLBCL is more common in middle-aged and elderly men, clinical manifestations lack specificity, first-line treatment is mainly surgery combined with standard chemotherapy regimens. The Lugano stage may be an independent prognostic factor affecting OS and PFS in PI-DLBCL patients.

## Introduction

Non-Hodgkin lymphoma (NHL) is a malignant tumor derived from immune cells. NHL is one of the most common hematolymphoid tumors worldwide, ranking eighth in the cause of death from cancer in the United States (1, 2). The gastrointestinal tract is the most frequent extranodal location of NHL. However, gastrointestinal lymphoma is a rare tumor, which accounts for only 10–15% of all NHL and 1–4% of gastrointestinal tumors (3, 4). Gastrointestinal lymphomas are mainly found in the stomach, followed by the small intestine and colorectum. Esophageal lymphomas are extremely rare, and the intestine is the second most commonly affected site after the stomach (5, 6). Primary intestinal lymphomas (PIL), including those of the small and large intestine, account for 20–30% of all gastrointestinal lymphomas, and the incidence is on the rise (1).

More than 50 different subtypes have been listed in the latest World Health Organization (WHO) Classification of Tumors of Hematopoietic and Lymphoid Tissues, follicular lymphoma is the most common indolent lymphoma, while diffuse large B-cell lymphoma is the most common aggressive NHL. Primary intestinal diffuse large B-cell lymphoma (PI-DLBCL) is malignancy that originates from the lymphocytes of the intestine. Diagnostic criteria of PI-DLBCL refer to Dawson’s diagnostic criteria (7). Most PI-DLBCL patients present with non-specific symptoms of the digestive system. The common diagnostic methods include ultrasound, contrast, CT, PET-CT, MRI, and endoscopy (8, 9). The treatment of PI-DLBCL include surgery, radiotherapy, chemotherapy and targeted therapy. Previous studies have shown that perforation, age ≥65 years, advanced stage, non-GCB type, EBER positivity, PD-L1 expression, and double expressor are prognostic factors in patients with PI-DLBCL (10, 11).

Nowadays, the academic research on PI-DLBCL is extremely limited, and there is no conclusion on the best treatment of PI-DLBCL. Therefore, the clinical data of 88 patients with PI-DLBCL were retrospectively analyzed in this study, and the latest literature was searched to systematically discuss the clinical characteristics, diagnosis and differentiation, treatment and prognosis of PI-DLBCL, so as to provide more basis for diagnosis and treatment of PI-DLBCL.

## Materials and methods

### Diagnostic method

In all patients included in this study pathological biopsy tissue was obtained at colonoscopy or abdominal surgery. All of them underwent bone marrow puncture biopsy after admission to determine the presence of bone marrow infiltration. Necessary laboratory tests were carried out. Comprehensive diagnosis of PI-DLBCL was performed after the above medical detection results were reported.

### Diagnostic criteria

Diagnostic criteria refer to Dawson’s diagnostic criteria, including: (1) unobserved superficial lymph nodes; (2) No mediastinal lymph node enlargement was found on chest radiographs; (3) Normal total white blood cell count; (4) Gastrointestinal tract and local lymph nodes were mainly involved, and no other lymph nodes were involved; (5) There was no evidence of liver and spleen involvement. Patients with elevated white blood cell counts due to neutrophilia but who meet all other Dawson criteria are also classified as PI-DLBCL.

### Inclusion criteria and exclusion criteria

Inclusion criteria: (1) confirmed by pathological biopsy according to Dawson's diagnostic criteria; (2) the lesion site was intestinal tract; (3) baseline assessment data and clinical data were detailed and complete.

Exclusion criteria: (1) patients with definite diagnosis but incomplete clinical data; (2) patients with DLBCL who have converted from other types of lymphoma; (3) had received antitumor therapy before enrollment; (4) combined with other uncured tumors; (5) patients with incomplete medical history, irregular diagnosis and treatment, or who give up treatment midway; (6) patients who do not agree to be included in this study due to privacy and other reasons.

### Therapeutic schedule (regimen)

The main first-line treatment was operation combined with chemotherapy. 8 patients (9.09%) received operation alone, 30 patients (34.09%) received chemotherapy alone, and 50 patients (56.82%) received combination therapy. There are total 80 patients with PI-DLBCL received chemotherapy at the beginning, among them, 55 patients were treated with R-CHOP regimen, 7 patients received CHOP regimen, 5 patients were treated with R-miniCHOP regimen, 3 patients received R-DAEPOCH regimen, 2 patients were treated with Chidamide combined with R-CHOP regimen, 2 patients were treated with R2-CHOP regimen, 2 patients received R-COP regimen, 4 patients received E-CHOP, CP, RDHAP, PD-1 combined with R-miniCHOP regimen each. Details of specific chemotherapy regimen and usage of chemotherapy drugs are shown in **Table 1**.

**Table 1:** Chemotherapy regimens and usage in 80 patients with PI-DLBCL received chemotherapy at the beginning.

Chemotherapy regimen	Specific types and usage of chemotherapy drugs
R-CHOP regimen	Rituximab (RTX) 375 mg/m^2^, intravenous d0; Cyclophosphamide (CTX) 750 mg/m^2^, intravenous d1; Doxorubicin (ADM) 40–50 mg/m^2^, intravenous d1; Vincristine (VCR) 1.4 mg/m^2^, intravenous d1; Prednisone (PED) 100mg, intravenous d1–5
CHOP regimen	CTX 750 mg/m^2^, intravenous d1; ADM 40–50 mg/m^2^, intravenous d1; VCR 1.4 mg/m^2^, intravenous d1; PED 100mg, intravenous d1–5
R-miniCHOP regimen	RTX 375 mg/m^2^, intravenous d0; CTX 400 mg/m^2^, intravenous d1; ADM 25 mg/m^2^, intravenous d1; VCR 1 mg/m^2^, intravenous d1; PED 40mg, intravenous d1–5
R-DAEPOCH regimen	RTX 375 mg/m^2^, intravenous d0; Etoposide (VP-16) 50mg/(m^2^·d), d1–4,96 hours continuous infusion; VCR 0.4mg/(m^2^·d), d1–4,96 hours continuous infusion; ADM 10mg/(m^2^·d), d1–4,96 hours continuous infusion; CTX 750mg/m^2^, d5; PED 60mg/(m^2^·d), d1–5
Chidamide combined with R-CHOP regimen	Chidamide 30mg, take orally, biw*; RTX 375 mg/m^2^, intravenous d0; CTX 750 mg/m^2^, intravenous d1; ADM 40–50 mg/m^2^, intravenous d1; VCR 1.4 mg/m^2^, intravenous d1; PED 100mg, intravenous d1–5
R2-CHOP regimen	Lenalidomide 25mg, take orally, d1–21; RTX 375 mg/m^2^, intravenous d0; CTX 750 mg/m^2^, intravenous d1; ADM 40–50 mg/m^2^, intravenous d1; VCR 1.4 mg/m^2^, intravenous d1; PED 100mg, intravenous d1–5
R-COP regimen	RTX 375 mg/m^2^, intravenous d0; CTX 750 mg/m^2^, intravenous d1; VCR 1.4 mg/m^2^, intravenous d1; PED 100mg, intravenous d1–5
E-CHOP regimen	VP-16 100mg/m^2^, intravenous d1–3; CTX 750 mg/m^2^, intravenous d1; ADM 40–50 mg/m^2^, intravenous d1; VCR 1.4 mg/m^2^, intravenous d1; PED 100mg, intravenous d1–5
CP regimen	CTX 750 mg/m^2^, intravenous d1; PED 100mg, intravenous d1–5
RDHAP regimen	RTX 375 mg/m^2^, intravenous d0; Dexamethasone (Dex) 40mg/d, intravenous d1–4; Cisplatin (DDP) 100mg/m^2^, 24 hours continuous infusion, d1; Cytarabine (Ara-C) 2g/m^2^, q12h*, d2
Sintilimab combined with R-miniCHOP regimen	Sintilimab 200mg, intravenous d0; RTX 375 mg/m^2^, intravenous d1; CTX 400 mg/m^2^, intravenous d2; ADM 25 mg/m^2^, intravenous d2; VCR 1 mg/m^2^, intravenous d2; PED 40mg, intravenous d2–6

biw, administer the medication twice a week; q12h, administer the medication every 12 hours.

### Curative effect evaluation

The 2014 edition of Lugano evaluation criteria was adopted for evaluation. Firstly, complete remission (CR): PET-CT has a Deauville score of 1–3 or CT examination showed that the lesion diameter was less than 1.5 cm. Secondly, partial remission (PR): PET-CT has a Deauville score of 4–5 associated with decreased metabolism from baseline or CT examination showed that the total PPD (long diameter times short diameter) of up to 6 target lesions was reduced by ≥50%.Thirdly, stable disease (SD): PET-CT has a Deauville score of 4–5,and there was no significant change in metabolism from baseline, or CT examination showed that the total PPD (long diameter times short diameter) of up to 6 target lesions was increased by <50%.Finally, progression disease (PD): PET-CT has a Deauville score of 4–5 associated with increased metabolism from baseline or new lesions with increased metabolism appear, or CT examination showed that PPD was increased by ≥50% or the length of the lesion was more than 1.5 cm in at least one lesion, or the length or short diameter of the lesion was increased by 0.5 cm (lesion ≤ 2 cm) or 1 cm (lesion > 2 cm) when it was the smallest in at least one lesion.

### Efficacy evaluation and follow-up

Follow-up data were obtained and recorded by consulting outpatient and inpatient medical records and telephone follow-up. The start time of observation was the date of the patient’s first diagnosis, and the end date of observation was set as May 30, 2023 or the patient’s death, and the end date of observation for the patients who were lost to follow-up was the date of the last follow-up. The follow-up frequency was to follow up the latest survival situation of patients once a month. Median follow-up was 72 (1–148) months. OS was the time from diagnosis to death or last follow-up, and PFS was the time from diagnosis to first recurrence or progression or death, both measured in months.

### Statistical analysis

IBM SPSS 26.0 statistical software was used for statistical analysis of the collected clinical data. Chi-square test was used for comparison between the classification data groups, the Kaplan−Meier method was used for survival analysis, univariate and multivariate analysis of prognostic factors was carried out. Survival curves were drawn with GraphPad Prism 9.0 software. The significance level was set at *P* < 0.05.

**Figure 1:**
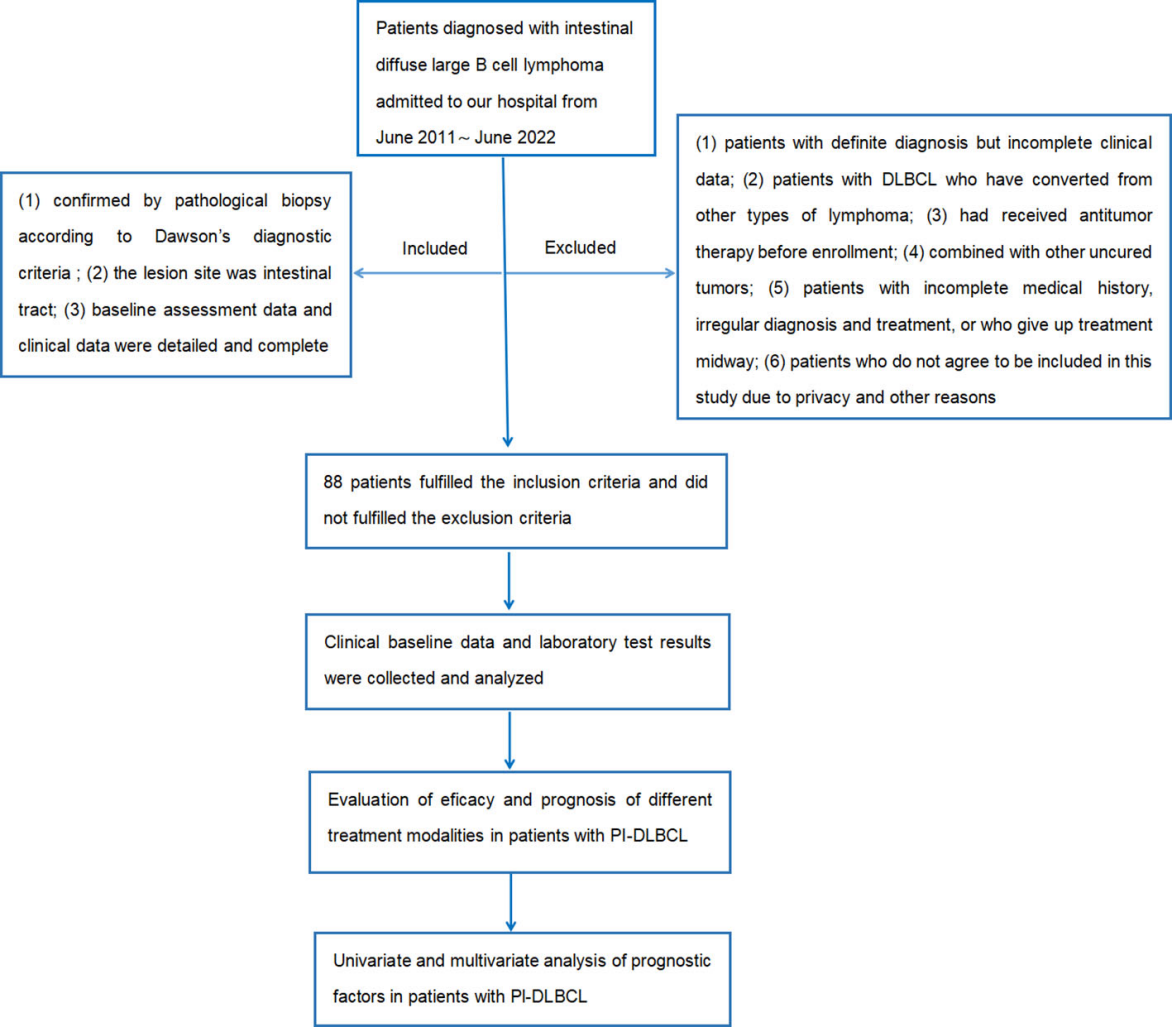
Flow chart of study design and screening sources.

The flow chart of the design and screening sources for this study is shown in **Figure 1**.

## Results

### Clinical data

In this study, the clinical data of 88 cases of patients with PI-DLBCL admitted to the Fujian Medical University Union Hospital from June 2011 to June 2022 were selected as the study objects. Among them, 60 were males and 28 were females. Age ranged from 18 to 82 years, with a median age of 57 years. Clinical baseline data such as age at first diagnosis, gender, ECOG score, Lugano stage, B symptoms, lymphoma site, pathological subtype, and IPI score of enrolled patients were collected and analyzed. Meanwhile, laboratory test results of leukocytes, serum lactate dehydrogenase, albumin, β_2_ microglobulin, Ki-67 and first-line treatment methods were collected and analyzed. The baseline data of all PI-DLBCL patients included in this study are shown in **Table 2**. This study was approved by the Medical Ethics Committee of Fujian Medical University Union Hospital (approval number: 2023KY060), and all patients and their families gave informed consent to this study.

**Table 2:** The baseline data of all PI-DLBCL patients.

Observed metrics	Number of cases (%)
Age(year)	
≤60	51 (57.95)
>60	37 (42.05)
Gender	
Male	60 (68.18)
Female	28 (31.82)
ECOG score	
< 2	45 (51.14)
≥2	43 (48.86)
Lugano staging	
Stage I-II	34 (38.64)
Stage III-IV	54 (61.36)
B symptoms	
Yes	36 (40.91)
No	52 (59.09)
Hans pathological classification	
GCB	35 (39.77)
Non-GCB	53 (60.23)
IPI score	
0–2	42 (47.73)
3–5	46 (52.27)
White blood cell count	
Normal	72 (81.82)
Abnormal	16 (18.18)
Serum LDH	
Normal	58 (65.91)
Elevated	30 (34.09)
Albumin	
≥35 g/L	58 (65.91)
<35 g/L	30 (34.09)
β2 Microglobulin	
≤3 μg/ml	67 (76.14)
>3 μg/ml	21 (23.86)
Ki-67(%)	
≥80%	66 (75.00)
<80%	22 (25.00)
Lymphoma site	
Small intestinal group	77 (87.50)
Non-small intestinal group	11 (12.50)
First-line treatment	
Surgical group	58 (65.91)
Non-surgical group	30 (34.09)

According to inclusion and exclusion criteria, combined with follow-up, a total of 88 patients were included in this study, of which 51 patients (57.95%) survived, 30 patients (34.09%) died, and 7 patients (7.95%) were lost to follow-up. There were 60 cases of males (68.18%) and 28 cases of females (31.82%), with a male to female ratio of 2.1:1. According to the Lugano stage, the clinical stage was mostly advanced (stage III-IV), with 33 patients (37.50%) of stage I-II and 55 patients (62.50%) of stage III-IV. The main complaint was abdominal pain in 62 patients (70.45%), bowel habit change in 16 patients (18.18%) and other complaints in 10 patients (11.37%). The median age of onset was 57 (17–82) years old. FISH analysis suggested double-hit DLBCL in 2 patients and tripple-hit DLBCL in 2 patients. The main first-line treatment was operation combined with chemotherapy. 8 patients (9.09%) received operation alone, 30 patients (34.09%) received chemotherapy alone, and 50 patients (56.82%) received combination therapy.

According to different first-line treatment methods, the two groups were divided into operation group (including operation alone and operation combined with chemotherapy) and non-operation group. Evaluation of efficacy after at least 2 courses of treatment, for PI-DLBCL patients (*P*=0.789), there was no statistically significant difference in the efficacy of surgery, and the results were shown in **Table 3**.

**Table 3:** Clinical efficacy and prognosis of different treatment modalities in patients with PI-DLBCL.

Features	Number of cases	Surgical group	Non-surgical group	*χ* ^2^ value	*P* value
Efficacy				0.072	0.789
CR+PR	57	37	20		
SD+PD	31	21	10		
Relapsed or refractory to treatment				0.008	0.927
Yes	24	16	8		
No	64	42	22		

**Figure 2:**
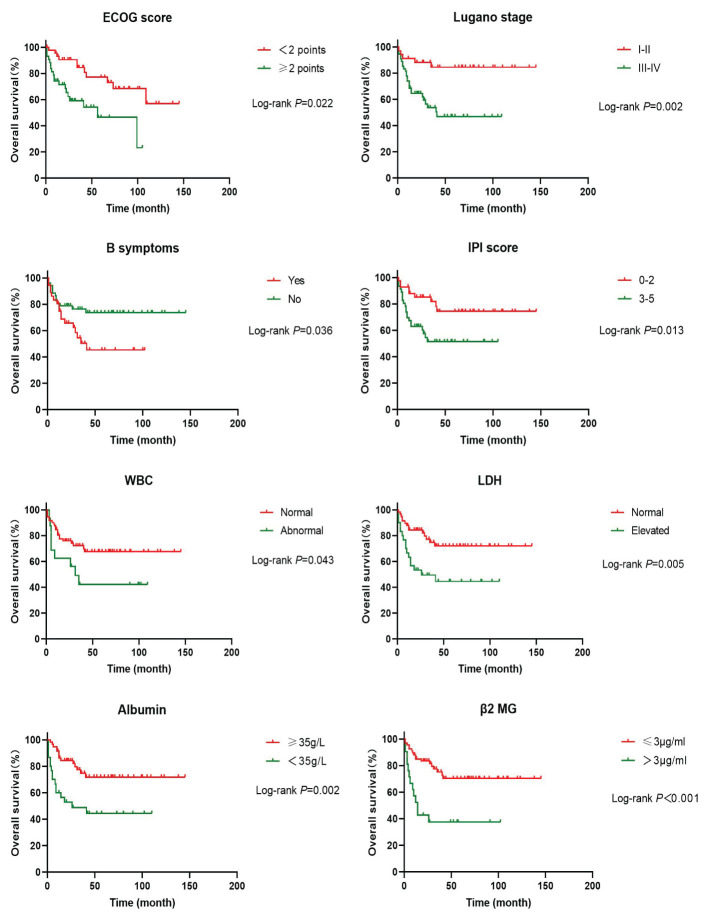
Kaplan-Meier overall survival curves for prognostic factors in PI-DLBCL patients.

**Figure 3:**
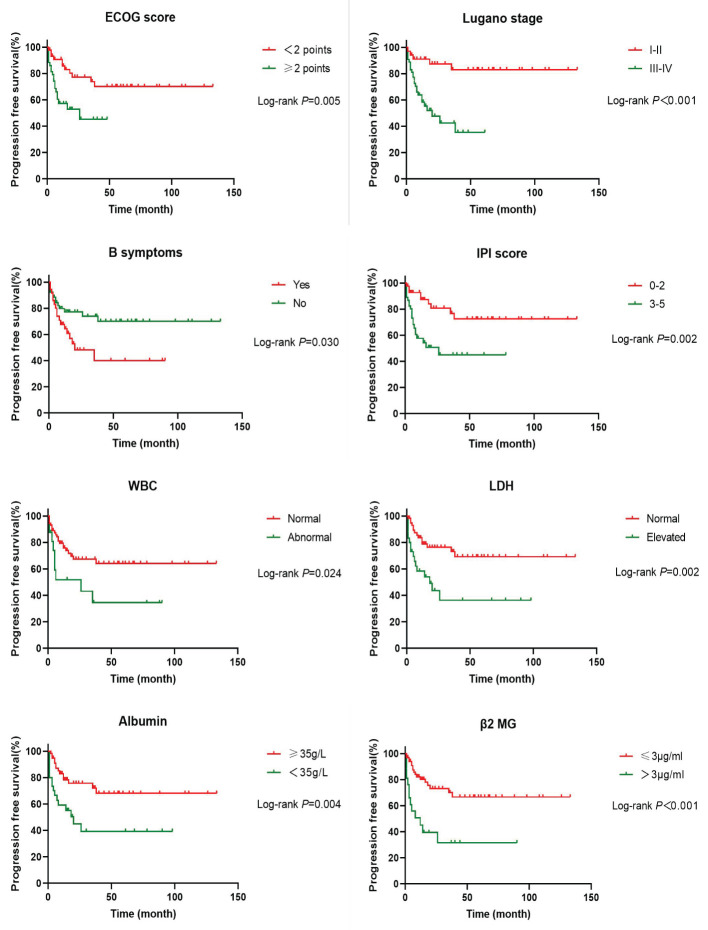
Kaplan-Meier progression free survival curves for prognostic factors in PI-DLBCL patients.

In order to investigate the effects of various influencing factors on the survival of 88 patients with PI-DLBCL, log-rank test was used for each indicator, and the results are shown in **Tables 4**, **5**. The results in the table showed that ECOG score, Lugano stage, B symptoms, IPI score, white blood cells, serum LDH, albumin, and β_2_ microglobulin had an impact on the OS of patients. ECOG score, Lugano stage, B symptom, IPI score, leukocyte, serum LDH, albumin and β_2_ microglobulin had influence on PFS of patients. The survival curve was drawn by Kaplan-Meier method, and the results were shown in **Figures 2**, **3**.

**Table 4:** Clinical features of PI-DLBCL and univariate analysis of OS.

Observed metrics	1 year OS (%)	3 years OS (%)	5 years OS (%)	*χ* ^2^ value	*P* value
Age(year)				0.436	0.509
≤60	78.30	67.70	64.10		
>60	75.70	63.10	59.60		
Gender				0.936	0.333
Male	73.30	60.30	60.30		
Female	85.70	77.70	67.30		
ECOG score				5.218	0.022
<2	86.60	79.00	72.60		
≥2	67.40	51.10	51.10		
Lugano stage				9.803	0.002
I-II	91.20	84.50	84.50		
III-IV	68.50	53.70	47.00		
B symptoms				4.408	0.036
Yes	74.80	50.40	45.30		
No	78.80	76.50	73.60		
Hans pathological classification				0.064	0.801
GCB	71.40	67.20	62.40		
Non-GCB	81.10	65.40	62.60		
IPI score				6.188	0.013
0–2	88.00	81.90	74.40		
3–5	67.40	51.50	51.50		
WBC				4.083	0.043
Normal	80.50	72.20	67.70		
Abnormal	62.50	42.20	42.20		
Serum LDH				8.065	0.005
Normal	84.40	74.80	72.00		
Elevated	63.30	49.50	44.60		
Albumin				9.647	0.002
≥35 g/L	86.10	74.80	71.90		
<35 g/L	60.00	48.90	44.40		
β_2_ Microglobulin				12.374	<0.001
≤3 μg/ml	85.00	75.20	70.30		
>3μg/ml	52.40	37.50	37.50		
Ki-67(%)				1.799	0.180
≥80%	74.20	63.20	58.50		
<80%	86.40	74.80	74.80		
Lymphoma site				1.291	0.256
Small intestinal group	75.30	62.50	60.50		
Non-small intestinal group	90.90	90.90	72.70		
First-line treatment				1.810	0.178
Surgical group	82.80	67.80	67.80		
Non-surgical group	66.70	62.50	51.10		

WBC, White blood cell count; Non-small intestinal group, Including large intestine group, large intestine and small intestine co-involved group.

**Table 5:** PI-DLBCL clinical features and PFS univariate analysis.

Observed metrics	1 year PFS (%)	3 years PFS (%)	5years PFS (%)	χ^2^ value	*P* value
Age(year)				0.191	0.662
≤60	72.60	61.00	61.00		
>60	71.00	59.50	59.50		
Gender				0.750	0.386
Male	68.20	57.50	57.50		
Female	79.90	68.00	60.50		
ECOG score				7.964	0.005
<2	85.70	73.80	70.10		
≥2	57.20	45.30	45.30		
Lugano stage				13.524	<0.001
I-II	91.10	83.10	83.10		
III-IV	58.40	42.40	35.40		
B symptoms				4.712	0.030
Yes	64.40	40.10	40.10		
No	77.30	73.90	70.00		
Hans pathological classification				0.216	0.642
GCB	69.20	62.90	55.90		
Non-GCB	74.00	59.70	59.70		
IPI score				9.479	0.002
0–2	87.30	76.80	72.50		
3–5	57.70	45.00	45.00		
WBC				5.066	0.024
Normal	75.90	67.40	64.20		
Abnormal	51.80	34.60	34.60		
Serum LDH				9.532	0.002
Normal	79.00	73.00	69.10		
Elevated	58.40	36.30	36.30		
Albumin				8.339	0.004
≥35 g/L	78.40	72.10	68.10		
<35 g/L	59.10	39.30	39.30		
β_2_ Microglobulin				12.839	<0.001
≤3 μg/ml	80.30	70.00	66.70		
>3 μg/ml	45.10	31.60	31.60		
Ki-67(%)				1.468	0.226
≥80%	69.30	56.30	53.20		
<80%	80.00	73.30	73.30		
Lymphoma site				1.450	0.228
Small intestinal group	69.10	56.30	56.30		
Non-small intestinal group	90.90	90.90	68.20		
First-line treatment				2.510	0.113
Surgical group	77.50	63.40	63.40		
Non-surgical group	60.50	55.40	47.50		

Age, sex, ECOG score, Lugano stage, B symptoms, pathological subtypes, IPI score, leukocyte, serum LDH, albumin, β_2_ microglobulin, Ki-67, lymphoma site, first-line treatment and other indicators of 88 PI-DLBCL patients were included in the *COX* regression model for OS univariate analysis. ECOG score, Lugano stage, B symptoms, IPI score, serum LDH, albumin, and β_2_ microglobulin were the influencing factors for OS in PI-DLBCL patients (**Table 6**). Variables with *P* < 0.20 in univariate *COX* regression analysis were included in the multivariate analysis model, and the results suggested that Lugano stage was an independent prognostic factor for OS in PI-DLBCL patients (**Table 7**).

**Table 6:** Univariate analysis of OS in PI-DLBCL patients with COX proportional hazards.

Index	*P* value	HR	95%CI	
			lower limit	upper limit
Age	0.514	0.787	0.384	1.614
Gender	0.340	0.674	0.300	1.515
ECOG score	0.028	2.316	1.097	4.888
Lugano stage	0.004	4.115	1.567	10.808
B symptoms	0.042	0.471	0.228	0.972
Hans pathological classification	0.802	0.911	0.439	1.892
IPI score	0.017	2.587	1.182	5.662
WBC	0.051	2.183	0.998	4.774
Serum LDH	0.007	2.697	1.315	5.534
Albumin	0.003	2.941	1.432	6.038
β_2_ microglobulin	0.001	3.379	1.635	6.986
Ki-67	0.191	0.526	0.201	1.376
Lymphoma site	0.272	0.448	0.107	1.880
First-line treatment	0.186	1.629	0.790	3.357

**Table 7:** Multivariate analysis of OS in PI-DLBCL patients with *COX* proportional hazards.

Index	*P* value	HR	95%CI	
			Lower limit	Upper limit
ECOG score	0.565	1.457	0.405	5.245
Lugano stage	0.038	3.757	1.078	13.100
B symptoms	0.185	0.596	0.277	1.281
IPI score	0.609	0.700	0.178	2.745
WBC	0.218	1.724	0.724	4.102
Serum LDH	0.749	1.178	0.432	3.209
Albumin	0.101	2.078	0.868	4.979
β_2_ microglobulin	0.502	1.439	0.498	4.156
Ki-67	0.560	0.702	0.214	2.306
First-line treatment	0.285	1.543	0.696	3.419

Age, sex, ECOG score, Lugano stage, B symptoms, pathological subtypes, IPI score, leukocyte, serum LDH, albumin, β_2_ microglobulin, Ki-67, lymphoma site, first-line treatment and other indicators of 88 PI-DLBCL patients were included in the *COX* regression model for PFS univariate analysis. ECOG score, Lugano stage, B symptoms, IPI score, leukocyte, serum LDH, albumin, and β_2_ microglobulin were the influencing factors for PFS in PI-DLBCL patients (**Table 8**). Variables with *P* < 0.20 in univariate *COX* regression analysis were included in the multivariate analysis model, and the results showed that Lugano stage was an independent prognostic factor affecting PFS in PI-DLBCL patients (**Table 9**).

**Table 8:** *COX* proportional hazards model for PFS univariate in PI-DLBCL patients.

Index	*P* value	HR	95%CI	
			Lower limit	Upper limit
Age	0.666	1.172	0.571	2.405
Gender	0.394	0.703	0.313	1.581
ECOG score	0.007	2.837	1.324	6.079
Lugano stage	0.001	5.203	1.954	13.852
B symptoms	0.036	0.459	0.222	0.952
Hans pathological classification	0.646	0.842	0.405	1.752
IPI score	0.004	3.222	1.457	7.125
WBC	0.031	2.369	1.082	5.188
Serum LDH	0.004	2.919	1.419	6.002
Albumin	0.006	2.731	1.330	5.606
β_2_ microglobulin	0.001	3.430	1.660	7.089
Ki-67	0.238	0.560	0.214	1.465
Lymphoma site	0.248	0.429	0.102	1.801
First-line treatment	0.122	1.769	0.858	3.647

**Table 9:** Multivariate analysis of PFS in PI-DLBCL patients with *COX* proportional hazards.

Index	*P* value	HR	95%CI	
			Lower limit	Upper limit
ECOG score	0.785	1.197	0.329	4.362
Lugano stage	0.009	5.772	1.553	21.457
B symptoms	0.222	0.625	0.294	1.329
IPI score	0.549	0.668	0.179	2.498
WBC	0.113	2.161	0.834	5.600
Serum LDH	0.356	1.561	0.607	4.015
Albumin	0.286	1.631	0.664	4.008
β_2_ microglobulin	0.517	1.390	0.513	3.767
First-line treatment	0.183	1.723	0.774	3.835

### Literature review

As shown in **Table 10**, the existing retrospective studies on PI-DLBCL from PubMed database in the past 10 years have been systematically listed and reviewed. The clinicopathological features and prognosis of PI-DLBCL patients in different regions were compared in various aspects. We can draw a conclusion that age, stage, treatment regimen, expression of certain genes, location of primary lesion and Epstein-Barr virus infection can seriously affect the prognosis of patients with PI-DLBCL. New prognostic models are emerging, and the best staging system is still under discussion.

**Table 10:** The existing published literature on PI-DLBCL.

Author	Year	Country	Number of cases	Research result	Research conclusion
Liu et al.	2022	China	24 cases	Age, marital status, Ann Arbor stage, surgery for primary site and chemotherapy are independent prognostic factors of OS for primary small intestinal DLBCL (PsI-DLBCL).	For PsI-DLBCL patients, a survival nomogram may be better at predicting OS.
Choi et al.	2022	Korea	37 cases	The prognosis of nonintestinal CD47-high DLBCL was poorer than that of intestinal CD47-high DLBCL. CD47-high DLBCL was closely associated with 18q21 gain/amplification and showed a high prevalence in intestine.	In patients with PI-DLBCL, high expression of CD47 suggests a better prognosis.
Zhang et al.	2021	China	22 cases	In elderly Chinese PI-DLBCL patients, surgery plus chemotherapy produced better results for event-free survival (EFS) than chemotherapy alone.	The treatment of PI-DLBCL in elderly Chinese patients is preferentially treated with surgery and chemotherapy.
Fan et al.	2021	China	184 cases	PI-DLBCL patients with an ileocecal lesion presented have better survival time than those with non-ileocecal sites, and patients received surgery with lymphadenectomy had the best OS and PFS.	PI-DLBCL patients with ileocecal lesion have a better prognosis, and surgery combined with lymphadenectomy is the preferred treatment option.
Wang et al.	2021	China	1602 cases	surgery significantly improved the survival rate of PI-DLBCL patients	Surgery is beneficial to survival in PI-DLBCL patients.
Wang et al.	2021	China	1613 cases	Visual prognostic model had better authentication capability than the International Prognostic Index (IPI) scoring model in the prediction of prognosis of PI-DLBCL.	Visual prognosis model can predict the prognosis of PI-DLBCL patients better than traditional methods.
Mou et al.	2021	China	23 cases	Ileocecal is the most primary site of intestinal DLBCL. Patients with DLBCL of the ileocecal region and small intestine except duodenum have low IPI and lactate dehydrogenase, high proportion of limited-stage tumors, high incidence of intestinal obstruction or perforation. The Epstein-Barr virus-encoded RNA-1 (EBER1) positive rate of duodenal DLBCL is higher.	The positive rate of EBER1 was higher in duodenum DLBCL, while in other small intestine DLBCL patients, IPI and LDH were low and the stages were mostly limited, but the incidence of intestinal obstruction or perforation was high.
Jiang et al.	2020	China	73 cases	High TLG, non-GCB and high NCCN-IPI are indicators of poor prognosis in PI-DLBCL patients.	The 18F−FDG PET/CT grading system can distinguish the prognosis of different PI-DLBCL patients.
Zhao et al.	2020	China	50 cases	R-CHOP immunochemotherapy plus surgery has a better prognosis than R-CHOP alone in Chinese PI-DLBCL population.	Surgery plays an important role in improving the prognosis of Chinese patients with PI-DLBCL.
Wang et al.	2019	China	68 cases	Bone marrow invasion is an independent risk factor of PFS in PI-DLBCL patients	Compared with other staging system, Lugano staging system for stratifying and predicting the prognosis of PI-DLBCL patients is better.
Ishikawa et al.	2018	Japan	62 cases	Programmed cell death ligand 1(PD-L1) and Epstein-Barr virus (EBV) positivity of tumor cells are poor independent prognostic factors for OS in patients with PI-DLBCL.	Patients with PI-DLBCL should be routinely evaluated for EBV and PD-L1 to better assess prognosis and select immune checkpoint inhibitors
Fujii et al.	2018	Japan	20 cases	Gene alteration of A20 did not affect the clinicopathological features of PI-DLBCL patients.	There were inconsistencies between gene deletion and protein expression in patients with PI-DLBCL.
Lu et al.	2016	China	59 cases	Compared with other region, PI-DLBCL in Taiwan carried a higher rate of perforation and EBV association.	PI-DLBCL patients should be careful to prevent intestinal perforation and screen for EBV infection.
Ikegami et al.	2016	Japan	33 cases	In PI-DLBCL patients, translocations involving immunoglobulin heavy chain gene (IGH) is an independent prognostic factor for better PFS.	PI-DLBCL patients with IGH translocation have a favorable prognosis.
Huang et al.	2015	China	54 cases	The prognosis of PI-DLBCL patients can be improved by surgery combined with chemoradiotherapy and rituximab	Patients with PI-DLBCL need comprehensive treatment

## Discussion

Primary gastrointestinal lymphoma is a common extranodal NHL, with an incidence of 0.5–1.1 per 100,000 people in developed countries. More than 60% of cases occur in the stomach, and the intestine is the second most commonly affected site (1, 2). As the most common type of NHL, DLBCL accounts for 30%-40% of all malignant lymphomas, and the median age of onset of DLBCL patients in China is about 10 years younger than patients in Japan and the United States (12). Most PI-DLBCL patients are not easy to diagnose clinically. At present, the research on PI-DLBCL is very limited. There are many prognostic factors for PI-DLBCL, but there is still no consensus on its treatment and prognosis assessment. In this study, the clinical data of 88 patients with PI-DLBCL was analyzed retrospectively and prognostic factors were identified in order to improve the understanding of PI-DLBCL.

As the etiology and pathogenesis of PI-DLBCL are still not yet clear, the existing research results suggest that the etiology of PI-DLBCL may be related to genetics, environmental factors, viral infection, immune deficiency, inflammatory bowel disease, organ transplantation and drugs (13–16). It is noteworthy that gut-associated lymphoid tissue (GALT) is mostly located in the ileocolonic region, which can produce specific immune responses, and repeated antigen stimulation of such lymphocytes can easily lead to monoclonal proliferation of this tissue and eventually it develops into lymphoma (16).

The results of our study showed that PI-DLBCL was more common in males (68.18%), and the median age of onset was 57 (17–82) years old. The most common and earliest clinical symptom was abdominal pain, which was consistent with the results reported in the literature (17, 18). The clinical manifestations of PI-DLBCL are often abdominal pain, ascites, hepatomegaly, splenomegaly, hemorrhage, general fatigue, anorexia, emaciation, and some patients may present with acute intestinal obstruction or perforation (19, 20). Since the lack of specificity of the above clinical manifestations, the diagnosis and treatment may be delayed and lead to poor prognosis. PI-DLBCL needs to be distinguished from intestinal ulcer, various inflammatory conditions, intestinal obstruction, intestinal carcinoma and other types of intestinal lymphoma (21–23). Although colon carcinoma is the most common intestinal malignancy, the possibility of intestinal lymphoma should still be considered in patients with recurrent abdominal pain. PI-DLBCL is rare, the initial symptoms are not obvious, the signs are not typical, and the patients are often in the advanced stage when diagnosed, so the early diagnosis is very challenging.

For the diagnosis of PI-DLBCL, the common imaging diagnostic methods include ultrasound, contrast, CT, PET-CT, MRI, and so on, as well as endoscopy such as gastroenteroscopy and capsule endoscopy. These methods should be used reasonably to improve the diagnosis rate of PI-DLBCL (24). Currently, one of the most recommended tests for lymphoma patients is PET-CT, which has irreplaceable advantages. In PI-DLBCL, PET-CT shows concentration of 18F-fluorodeoxyglucose metabolism (18F-FDG) at the lesion, and the SUV value fluctuate between 3.6–33.7, which could accurately locate the enteric and external lesions of PI-DLBCL. In addition to emergency surgery, endoscopy is an important tool for the diagnosis of intestinal lymphoma. Endoscopy can visually inspect the entire diseased intestine, locate the specific location of the lesion, and biopsy can be performed to enable the correct preoperative diagnosis of PI-DLBCL. In addition, it can visually compare the lesions in the same site before and after treatment. This method can produce less trauma and lower cost, and is worth advocating and applying (25). In PI-DLBCL patients, ESR, LDH, β_2_-MG and other laboratory test indicators can be elevated, and Ki-67 index >90% is seen in most PI-DLBCL patients. When the bone marrow of patients with PI-DLBCL is invaded, it can cause anemia, thrombocytopenia, leukopenia or abnormal elevation (26).

Due to the high clinical diversity of intestinal DLBCL patients, the gold standard for diagnosis is histopathological biopsy, and the treatment methods include surgery, chemotherapy and immunotherapy, among which the standard first-line chemotherapy regimen is R-CHOP (rituximab combined with cyclophosphamide, Epirubicin, vindesin, dexamethasone) (27). In our study, we found that surgery is a commonly used method in the diagnosis and treatment of PI-DLBCL. Complete resection of the lesion can not only relieve the symptoms of the patient, but also reduces the risk of complications such as perforation, bleeding, obstruction or intussusception. In addition, the early pathological diagnosis and clinical staging of the biopsy tissue obtained by surgery are of guiding significance for clinical diagnosis and treatment, and will not increase the risk of death of the patient (28). Wang et al. (29) found that patients with PI-DLBCL whose lesion site was in the small intestine had a reasonable survival prognosis after surgery, and there was no difference in treatment and prognosis. Since PI-DLBCL is usually highly malignant, surgical treatment alone is not recommended. Patients with PI-DLBCL may benefit from surgery combined with chemotherapy and immunotherapy. The results of this study suggest that surgery in PI-DLBCL patients had no statistical significance in OS and PFS, which was considered to be related to the small number of study cases and could not represent the whole population.

At present, the academic consensus for the treatment of PI-DLBCL is that surgery combined with adjuvant therapy, including radiotherapy, chemotherapy and targeted therapy, can result in a better prognosis (30). However, previous studies have found that the intestine is less suitable for radiation therapy than the stomach, and chemotherapy for primary small intestinal lymphoma appears to have a survival benefit over surgery alone, while colon lymphoma favors surgery plus chemotherapy (31). Chemotherapy for NHL in the small intestine occasionally causes perforation, which is life-threatening during chemotherapy. Perforation occurs in about 9% of patients with gastrointestinal lymphoma. The risk of perforation in aggressive lymphomas is more than 6 times greater than in indolent B-cell lymphomas, and perforation occurs due to transmural damage caused by tumor or tissue necrosis after chemotherapy. A 37-year retrospective study of patients with gastrointestinal lymphoma found that 9% developed perforation, while 55% of perforations occurred after chemotherapy. The most common perforation site was the small intestine (59%), followed by the large intestine (22%) and stomach (16%) (32). Last year, a study found that DLBCL in the ileocecal area showed a characteristic mutation pattern with the most frequent TP53 mutation (52.6%) and 18q21 gain (42.1%). This provides more evidence for targeted therapy of PI-DLBCL (33). In addition, studies have also shown that surgical treatment of primary intestinal NHL before chemotherapy can prevent perforation and reduce tumor. Wong et al. (34) confirmed that total parenteral nutrition (TPN) and fasting could not reduce the risk of perforation in PIL patients receiving chemotherapy. Clinically, this complication should be considered in advance and preventive measures should be taken according to the degree of tumor burden (35).

Studies have suggested that perforation, age ≥65 years, advanced stage, non-GCB type, EBER positivity, PD-L1 expression, and double expressor NHL are associated with poor prognosis in patients with PI-DLBCL (36, 37). The patients with EBV+ PI-DLBCL showed a progressive disease despite receiving R-containing chemotherapy and underwent an autologous peripheral blood stem cell transplantation (38). Double-hit and triple-hit genetics are associated with lower rates of complete remission (CR) and, consequently, in poor OS in DLBCL patients. Therefore, patients with double-hit and triple-hit NHL need aggressive interventions (39, 40). The results of univariate *Cox* regression analysis in this study showed that ECOG score, Lugano stage, B symptoms, IPI score, serum LDH, albumin, β_2_ microglobulin were the influencing factors of OS in PI-DLBCL patients, and ECOG score, Lugano stage, B symptoms, IPI score, white blood cells, serum LDH, albumin, β_2_ microglobulin were all the influencing factors of PFS in PI-DLBCL patients. It has been reported in the literature that the main affected sites and treatment methods do not affect the prognosis of patients with B-cell PIL (41), and the results of this study are consistent with those reported in the literature. Multivariate *Cox* analysis results of this study showed that Lugano stage is an independent prognostic factor affecting OS and PFS in PI-DLBCL patients, indicating that Lugano stage was an important prognostic factor, which was consistent with literature reports (41, 42). Some studies suggested that intestinal DLBCL with expression of CD47 have a better prognosis than that without CD47 expression (43). A study on adult PI-DLBCL showed that 3-, 5-, and 10-year survival rates in the whole cohort were 68.7%, 62.4% and 50.2% (29), while another study about intestinal FL showed that 3-year PFS and 3-year OS were 70% and 100% respectively, 5-year PFS and OS were 86.3% and 100% respectively (44).

To sum up, PI-DLBCL patients are mostly middle-aged and elderly men, which early clinical manifestations, symptoms and signs lack specificity. Imaging and laboratory examinations need to be comprehensive to identify the lesions and the extent of systemic involvement, and pathology is the gold standard for diagnosis. The first-line treatment of PI-DLBCL is mainly surgery combined with chemotherapy. Lugano stage is an independent prognostic factor affecting OS and PFS in PI-DLBCL patients. In the future, this research group will cooperate with multi-center units to further explore and study the clinical characteristics, diagnosis and treatment experience of rare extranodal lymphoma.

## Data Availability

The original contributions presented in the study are included in the article/supplementary material. Further inquiries can be directed to the corresponding author.
